# Use of Closed Incision Management with Negative Pressure Therapy for Complex Cardiac Patients

**DOI:** 10.7759/cureus.506

**Published:** 2016-02-23

**Authors:** V Sreenath (Seenu) Reddy

**Affiliations:** 1 Director of CV Surgery Outreach & Structural Heart Program, Centennial Heart and Vascular Center

**Keywords:** prevena, negative pressure wound therapy, npwt, sternal wound infections, sternal dehiscence

## Abstract

Background: In patients with major comorbidities undergoing complex cardiothoracic surgery, incision management is critical. This retrospective review evaluated negative pressure over closed sternal incisions in cardiac patients with multiple comorbidities within 30 days post-median sternotomy.

Methods: Records of post-sternotomy patients treated with Prevena™ Incision Management System (KCI, an Acelity company, San Antonio, TX), a closed incision negative pressure therapy (ciNPT), were reviewed from September 2010 through September 2014. Data collected included demographics, major comorbidities, types of surgery, relevant medical history, incision length, therapy duration, time to follow-up, and incision complications. Descriptive statistics were computed for continuous variables, frequency, and percentages for categorical variables.

Results: Twenty-seven patients were treated with ciNPT between September 2010 and September 2014. The mean patient age was 62.5 (SD 7.9), and the mean body mass index (BMI) was 38.5 (SD 4.4) kg/m^2^. Risk factors included obesity (BMI ≥ 30 kg/m^2^, 27/27; 100%), diabetes (25/27; 92.6%), hypertension (16/27; 59.3%), and 20/27 patients (74%) had ≥ 5 comorbidities. Mean ciNPT duration was 5.6 (SD 0.9) days. Within 30 days post-surgery, 21/27 (77.8%) patients had intact incisions with good reapproximation. Two patients experienced minor dehiscences; four cases of superficial cellulitis were treated and resolved. One patient with a dehiscence was readmitted for intravenous antibiotics and five patients were managed successfully with antibiotics as outpatients. All patients had intact incisions with good skin approximation at final follow-up.

Conclusions: In this retrospective study of post-sternotomy patients at high risk of developing complications, ciNPT over closed sternal incisions resulted in favorable outcomes within 30 days of surgery.

## Introduction

Approximately 0.3 - 5% of median sternotomy incisions are affected by complications, such as infection and dehiscence [1]. Compromised sternal incision healing has been associated with prolonged postoperative recovery and increased mortality [2]. The US Center for Disease Control and Prevention requires surveillance programs that monitor the development of surgical site infections within the first 30 days post-surgery for specific types of surgery [3]. Implementation of strategies to reduce incision complications is a critical concern of physicians treating patients who have undergone complex cardiothoracic procedures.

One strategy is the identification of patient characteristics and medical procedures associated with increased risk of incision breakdown. Diabetes [4-7], obesity [5-9], and advanced age [7, 10] have been reported as indicators of potential sternal incision complications. Chronic obstructive pulmonary disease (COPD) [4, 9-10], hypertension [6], reduced ejection fraction [5, 11], and complex or combined operative procedures [6] have also been associated with an increased likelihood of complications.

A variety of sternal incision management strategies have been reported in the literature, including antimicrobial-impregnated gauze [12], triclosan-coated sutures [13], cyanoacrylate-sealed Donati sutures [14], muscle flaps [15-16], and thorax support vests [17-18]. Several recent studies have reported positive outcomes using the Prevena™ Incision Management System (KCI, San Antonio, TX), a closed incision negative pressure therapy (ciNPT), over closed sternal incisions [18-20].

Our retrospective chart review evaluated the 30-day and follow-up results of complex cardiac patients whose post-sternotomy closed incisions were managed with ciNPT.

## Materials and methods

Data collection adhered to institutional guidelines for use of de-identified patient data, including patient consent for possible use of pictures without identifying marks/facial features. The data pool consisted of cardiac patients treated by the same surgeon at two institutions (Centennial Heart and Vascular Center, Nashville, TN and University Hospital, San Antonio, TX) from September 2010 through September 2014. Collected data included demographics (gender, age, and body mass index [BMI]), comorbidities, types of surgery, relevant medical history, the length of the incision, duration of therapy, time to follow-up, and types and numbers of complications. 

Per the Surgical Care Improvement Project protocol guidelines, patients received antibiotics within 30 minutes of the incision, at the four-hour intraoperative mark, and for up to, but not exceeding, 24 hours postoperatively [21]. The multiple layer closure technique consisted of running Vicryl^®^ sutures (Ethicon US LLC, Somerville, NJ) for deep layers, smaller Vicryl^®^ sutures for subcutaneous layers, and 4-0 Monocryl^®^ sutures (Ethicon US LLC, Somerville, NJ) for skin.

All closed sternal incisions were managed with ciNPT, using a portable, disposable negative pressure therapy unit and a Prevena™ Peel and Place Dressing™ (KCI, San Antonio, TX), a pre-sized foam dressing encapsulated in a polyurethane shell. According to institutional guidelines, patients treated with ciNPT had to have at least three of the following indications: BMI ≥ 35 kg/m^2^, diabetes, low albumin, poor tissue quality at the time of incision, recent postoperative infection, steroid exposure (either currently or within one week of use), or high dosage of pressor medication.

In all patients, the surgeon applied the ciNPT dressing over the closed incision, when the skin was cleaned and dried but still within the intact sterile field. Occasionally Mastisol^®^ Liquid Adhesive (Ferndale Laboratories, Inc., Ferndale, MI) was used as an additional adhesive for the dressing border tape, especially when the surface was uneven. The unit provided a continuous negative pressure of ‑125 mmHg. Placement of mediastinal and pleural drains slightly more inferior and lateral to the incision than usual and application of additional drape strips facilitated a better seal for the ciNPT dressing.

Computed descriptive statistics included mean, standard deviation, median, minimum, and maximum for continuous variables, and frequency and percentages for categorical variables. All analyses were performed using Statistical Analysis System version 9.3 (SAS^®^ software; SAS^®^ Institute Inc., Cary, NC).

## Results

In this group of 27 cardiac patients treated with ciNPT, the mean age was 62.5 (standard deviation [SD]: 7.9). All patients had ≥ 2 comorbidities, and 20/27 (74%) had ≥ 5 comorbidities (Table 1). All patients (27/27; 100%) were obese (BMI ≥ 30 kg/m^2^). Categorized according to clinical definitions of obesity [22-23], five patients were obese (BMI: 30‑34 kg/m^2^), 12 were severely obese (BMI: 35-39 kg/m^2^), nine were morbidly obese (BMI: 40-50 kg/m^2^), and one was super obese (BMI: > 50 kg/m^2^). The mean BMI was 38.5 kg/m^2^ (SD: 4.4). Almost all patients (25/27; 92.6%) had diabetes. Approximately half of the patients had poor nutritional status indicated by low albumin levels (13/27; 48.1%) and poor tissue quality (16/27; 59.3%).

**Table 1:** Patient Comorbidities

Comorbidity	N=27 n (%)
Obesity	27 (100.0%)
Diabetes	25 (92.6%)
Chronic Obstructive Pulmonary Disease	8 (29.6%)
Sleep Apnea	10 (37.0%)
Degenerative Joint Disease/ Osteoporosis	6 (22.2%)
Hypertension	16 (59.3%)
Hyperlipidemia	2 (7.4%)
Low Ejection Fraction	7 (25.9%)
Low Albumin	13 (48.1%)
Tobacco Abuse	5 (18.5%)
Poor Tissue	16 (59.3%)
Anemia	8 (29.6%)

The surgical procedures performed on these patients are shown in Table 2. Approximately half of the patients (14/27; 51.9%) underwent coronary artery bypass grafting (CABG) with ≥ 2 grafts. The mean incision length was 17.4 cm (SD: 1.6); mean duration of ciNPT was 5.6 days (SD: 0.9).

**Table 2:** Types of Surgical Procedures

Surgical Procedure	N = 27 n (%)
AVR Only	1 (3.7%)
MVR Only	1 (3.7%)
Sternal Reconstruction	4 (14.8%)
AVR and CABG	3 (11.1%)
MVR and CABG	2 (7.4%)
CABG with Maze procedure	2 (7.4%)
CABG with ≥2 grafts	14 (51.9%)
AVR - aortic valve replacement; MVR - mitral valve replacement; CABG, coronary artery bypass grafting	

Within the first 30 days post-surgery, 21/27 (77.8%) of patients had intact incisions that showed good reapproximation of the skin. During that time period, two patients experienced minor skin dehiscences and four patients had superficial cellulitis. One patient with a dehiscence was readmitted for intravenous antibiotics; the other five patients received treatment on an outpatient basis. On postoperative day 35, one patient presented with a sternal wound infection that resolved with local wound care and antibiotics. The mean follow-up was 6.7 weeks (SD: 3.1), and by final follow-up, all incisions were intact with good skin approximation. 

### Case study 1

**Figure 1:**
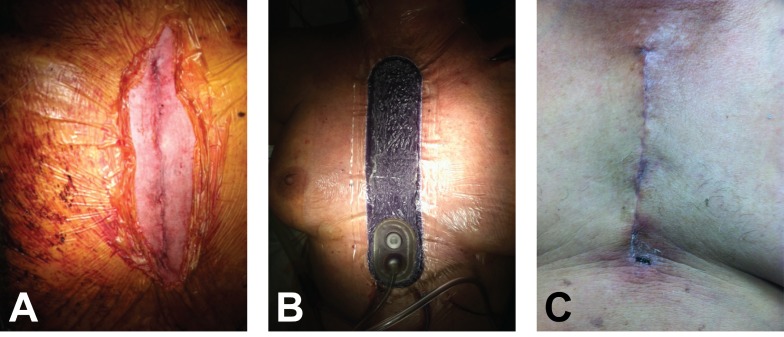
A 67-year-old woman, who underwent aortic valve replacement and coronary artery bypass grafting, had closed incision negative pressure therapy (ciNPT) placed over the incision. A) Clean closed 15 cm incision. B) ciNPT dressing in place with negative pressure applied. C) Incision was well approximated on postoperative day 10.

A 67-year-old woman presented with dyspnea on exertion and angina with activity. Her comorbidities included diabetes, obesity, sleep apnea, degenerative joint disease, osteoporosis, hypertension, and hyperlipidemia. Her medical history included previous cholecystectomy and myocardial infarction. Labs revealed a creatinine level of 1.6 mg/dL, chronic kidney disease (Stage III), and a normal hematocrit. Post-aortic valve replacement (AVR) and CABG, ciNPT was placed over the closed incision for 5.5 days (Figures 1A-1B). On postoperative day 10, incision edges were well approximated (Figure 1C). At the 13-week follow-up, the incision remained intact with good reapproximation.

### Case study 2

**Figure 2:**
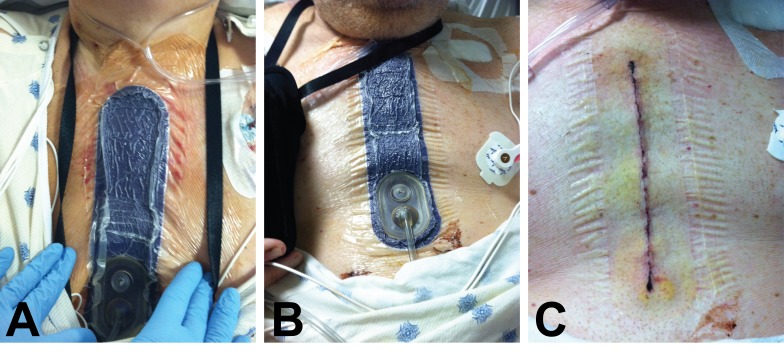
A 64-year-old male with multivessel artery disease and acute myocardial infarction had aortic valve replacement followed by closed incision negative pressure therapy (ciNPT) over the incision. A) Placement of ciNPT over the patient’s 17 cm incision. B) ciNPT was applied for 5 days. C) Incision edges were well-approximated at dressing removal postoperative day 5.

A 64-year-old male presented with dyspnea on exertion and angina with minimal activity. Patient comorbidities included diabetes, obesity, hypertension, hyperlipidemia, and COPD. Medical history reported severe, poorly controlled diabetes (HbA1c of 8), poor nutrition with low preoperative albumin (3.1), and appearance older than his age. Patient’s diagnoses on admission included multivessel artery disease and acute myocardial infarction. Treatment consisted of an AVR followed by ciNPT on the closed incision (Figures 2A-2B). On postoperative day 5, incision edges were well approximated after removal of the ciNPT (Figure 2C). At the 12-week follow-up, the incision was intact with good reapproximation.

## Discussion

This retrospective review evaluated ciNPT over the post-sternotomy incisions of 27 cardiac patients with multiple comorbidities, including obesity, diabetes, COPD, low albumin, and hypertension. These risk factors and extensive surgical procedures increased likelihood of sternal incision breakdown, warranting ciNPT application over the closed incisions. Within the first 30 days post-surgery, the majority of patients (77.8%) had intact incisions and no major sternal complications.

This patient population could be considered among the most critically-ill patients. All patients were obese and more than 90% were diabetic. The majority of patients also had other well-defined risk factors (e.g., COPD, hypertension, low albumin, and poor tissue quality). Both diabetes [4-7, 11, 24] and obesity [5-9, 11, 24] have been identified as independent risk factors for impaired sternal wound healing. Obesity contributes to dehiscence by exerting additional stress on the closed incision, thereby producing micro-separations in the skin that allow the ingress of skin flora [18, 25]. The relative avascularity of adipose tissue, which is organized in fat lobules, provides a receptive environment for bacteria, increasing the likelihood of infection, which can lead to dehiscence [26-27]. The degree of obesity also increases the risk level. Karunakar, et al. reported in their retrospective study that morbidly obese patients (BMI ≥ 40 kg/m^2^) were five times more likely to have a wound infection [28]. The retrospective study by Hysi, et al. noted that severely obese diabetic patients had significantly higher rates of mediastinitis *(p *= 0.002) and superficial wound infection *(p *= 0.003) [29]. Most of our patients were both obese and diabetic. With ciNPT, there were no documented cases of deep wound infections and the cases of mild cellulitis resolved without incision breakdown. The two superficial dehiscences that occurred within 30 days post-surgery were treated and remained intact at the time of final follow-up.

Extensive surgical procedures constituted a further risk factor. In a literature review of factors and scales for predicting the likelihood of deep sternal wound infection, Buja, et al. identified the duration of an operation as a surgical operative risk factor [30]. Matros, et al. conducted a retrospective study of all median sternotomies at their institution from 1991 through 2006 and stated that prolonged bypass time was the only independent variable associated with deep sternal wound infection [31].

Initial preclinical studies investigated the science behind ciNPT to better understand the mechanisms of action involved. Computer finite element analyses, as well as benchtop studies, indicated that ciNPT helped to hold incision edges together and protected incisions from external contamination [32]. Studies also showed that ciNPT removed fluid and infectious materials.

Studies using negative pressure over closed sternal incisions have reported favorable clinical outcomes. In a 2009 retrospective study, Atkins, et al. reported the use of NPWT (V.A.C.^®^ Therapy, KCI, San Antonio, TX) over the closed sternal incisions of 57 adult cardiac patients at high risk of infection [2]. In this study, 29 (50.9%) patients were both obese (BMI: 35.3 ± 6.7) and diabetic. Although at least three sternal wound infections were expected, none occurred [2]. In 2011, Colli, et al. evaluated ciNPT use over the closed sternal incisions of 10 patients with a mean Fowler risk score of 15.1. There were no cases of infection and no device- or wound-related complications [19]. Grauhan, et al. reported using ciNPT in a 2013 prospective study that compared 150 consecutive obese patients (BMI ≥ 30) whose wounds were treated with either ciNPT (n=75) or conventional sterile dressings (control, n=75) [18]. Patients treated with ciNPT had fewer wound infections within 90 days: 3/75 (4%) versus 12/75 (16%), respectively; *p *= 0.0266. The one ciNPT superficial infection required debridement and secondary wound closure as did four of the 10 control superficial infections [18]. For the majority of high-risk patients in our retrospective study, ciNPT resulted in intact incisions within the first 30 days post-surgery. None of our patients required the more extensive debridement and secondary wound closure reported in the Grauhan study.

Unlike the 90-day primary endpoint of the prospective Grauhan study, our retrospective study focused on the 30-day results and demonstrated favorable initial experience using ciNPT to manage sternal incisions. Limitations included small group size, as well as the retrospective and non-comparative nature of the study.

Our positive results from using ciNPT in patients that were at high-risk for incision breakdown were similar to those reported in larger studies using negative pressure over closed incisions following lower extremity fractures [33], total hip arthroplasty [34], hernioplasty [35], abdominoperineal resection [36], vascular surgery [37], and cesarean sections [38]. Given the multiple comorbidities of these patients and the complex nature and/or combination of the surgical procedures, ciNPT provided an alternative to conventional dressings, such as gauze dressings [12], topical dermal adhesives [39], or paper tape [40]. The ability to leave the ciNPT dressing undisturbed for up to seven days has also been reported to help protect incisions from external contamination and maintain reapproximation of wound edges against added tension due to patient obesity [18]. 

## Conclusions

In this retrospective study of post-sternotomy patients at high risk of developing complications, ciNPT over closed sternal incisions resulted in favorable outcomes within 30 days of surgery. Larger prospective comparative studies evaluating the 30-day efficacy of ciNPT over closed sternal incisions are warranted.
